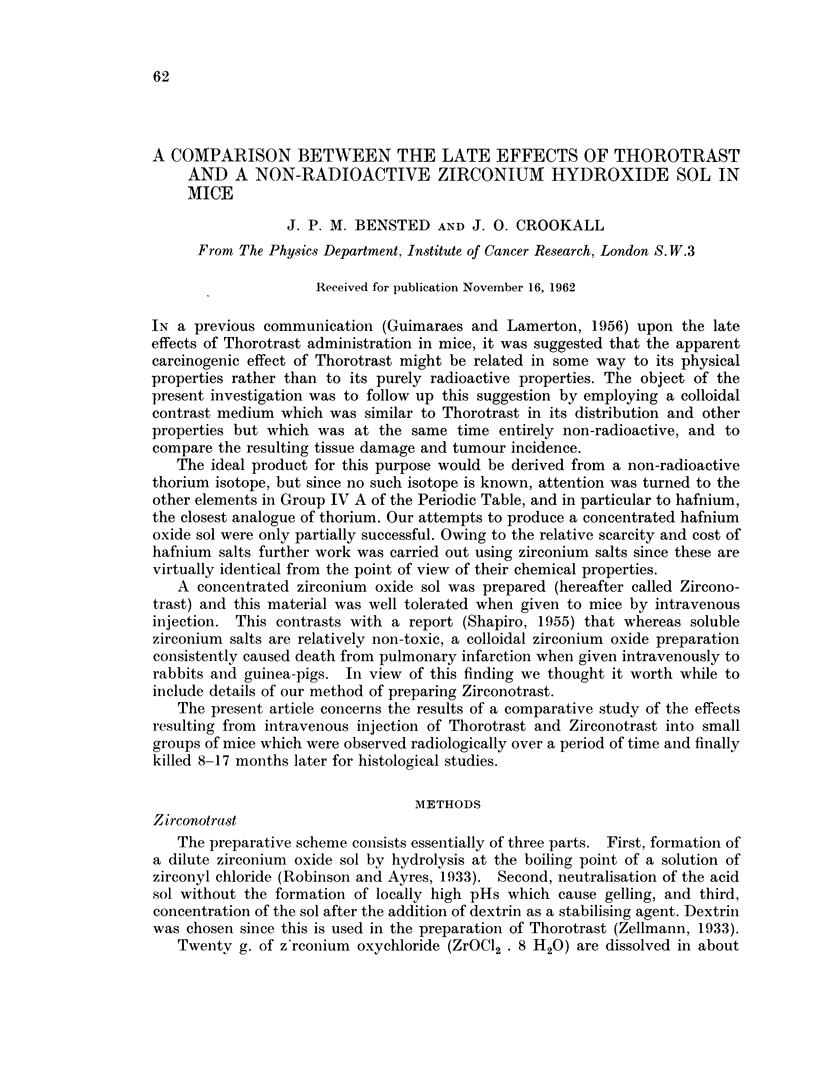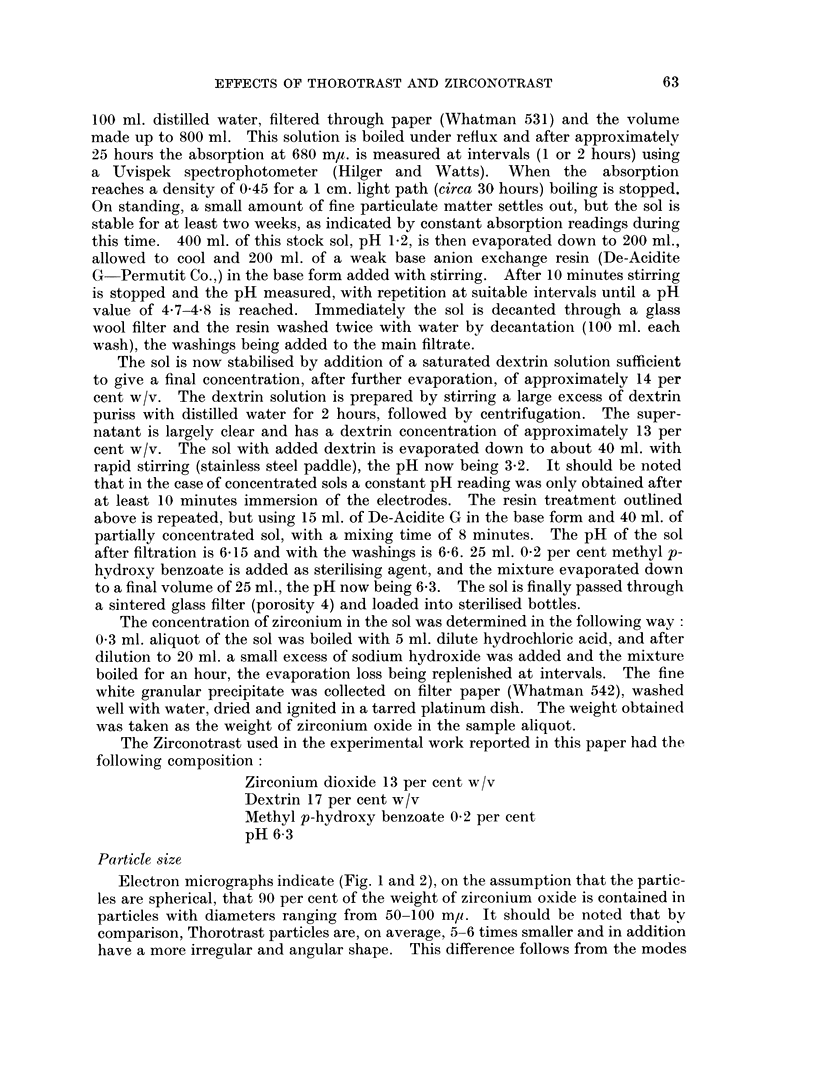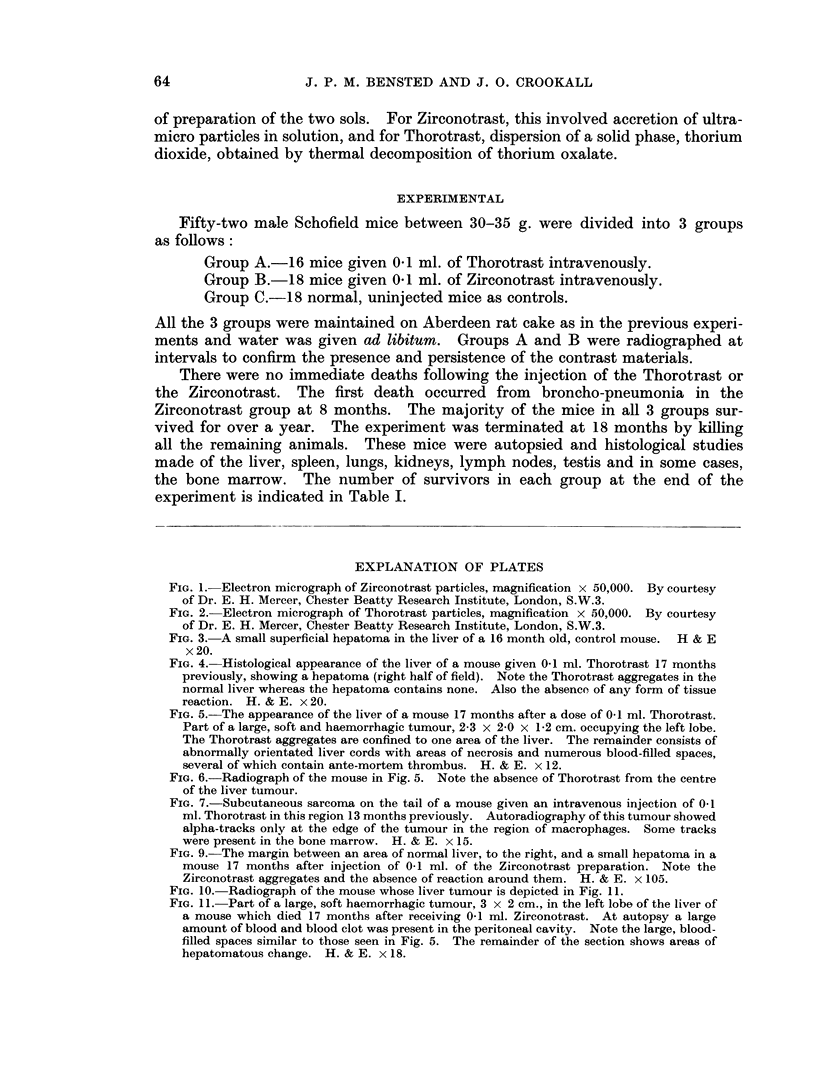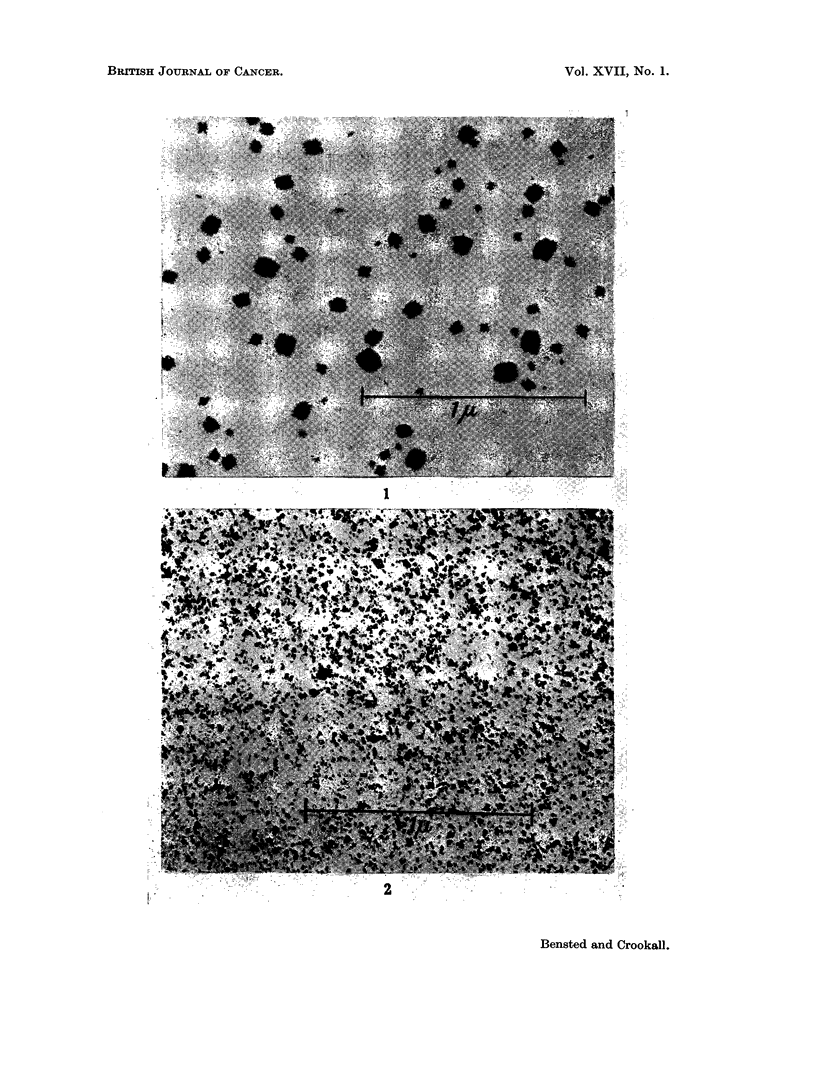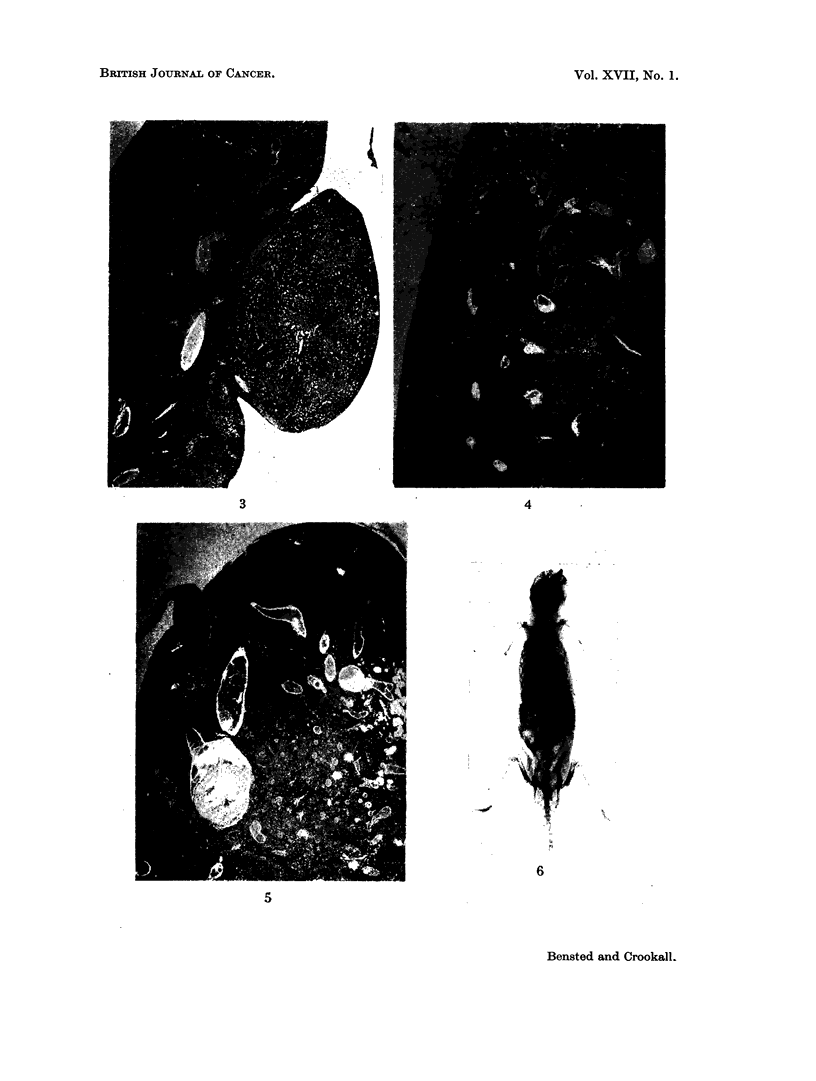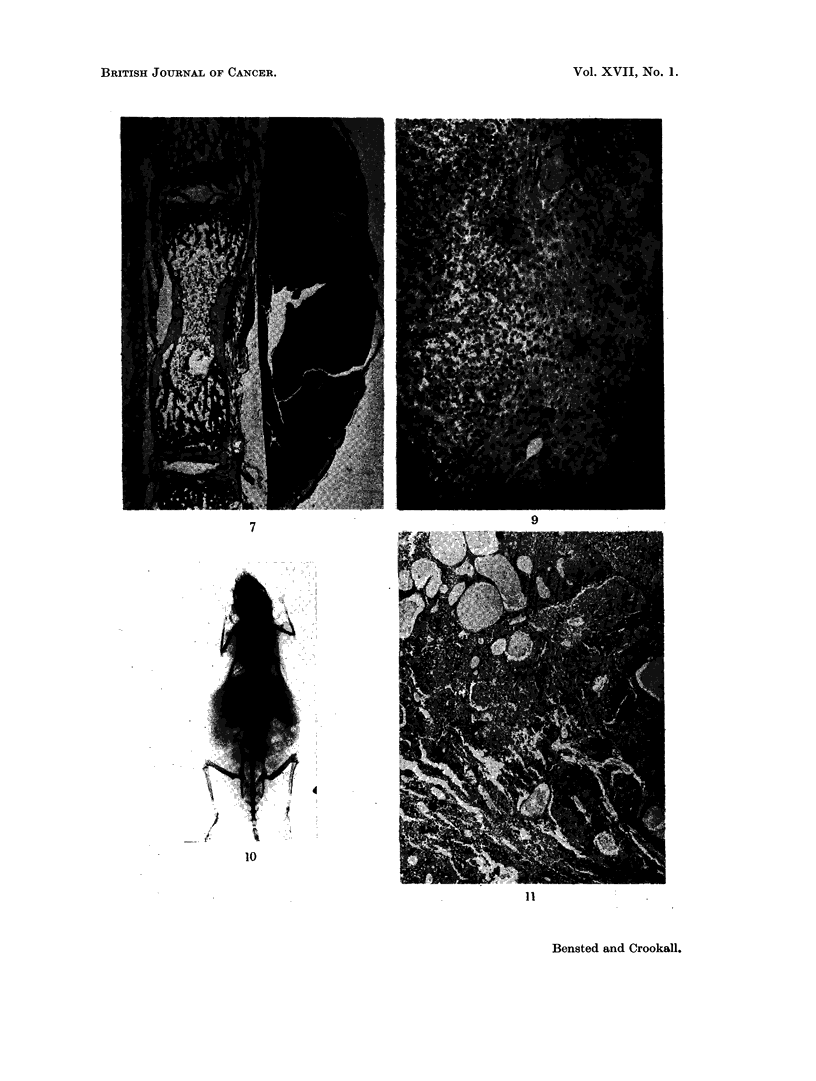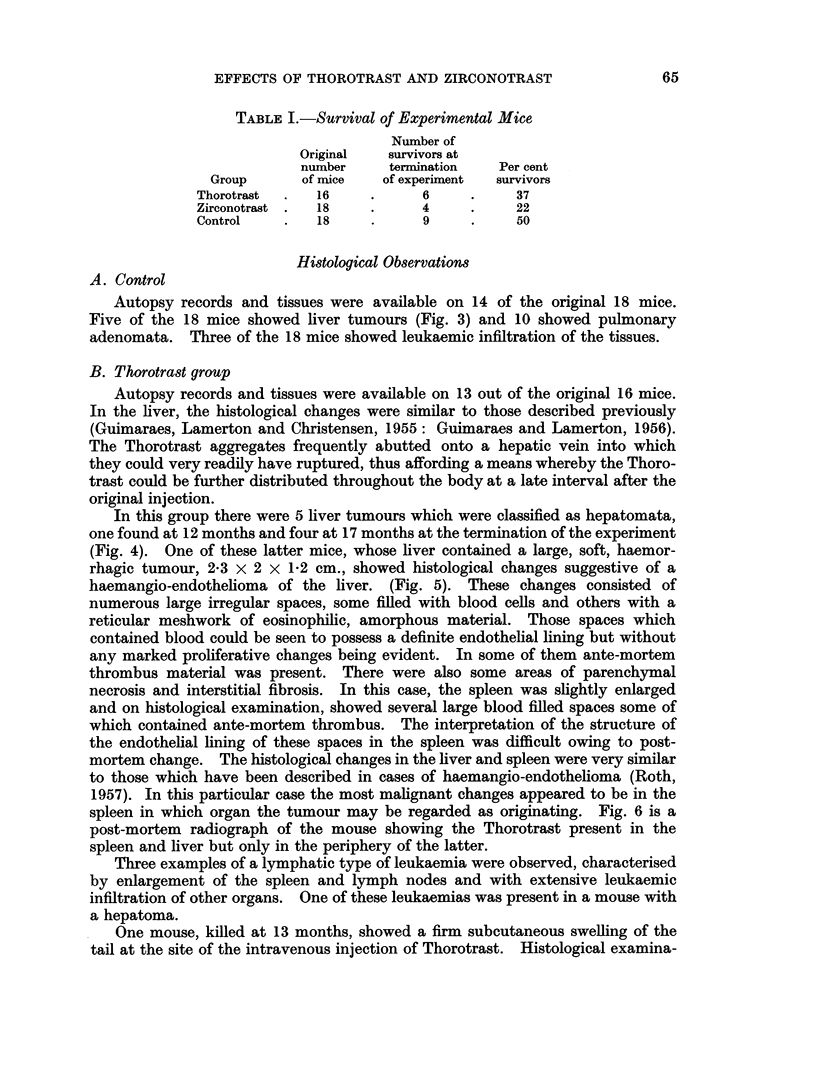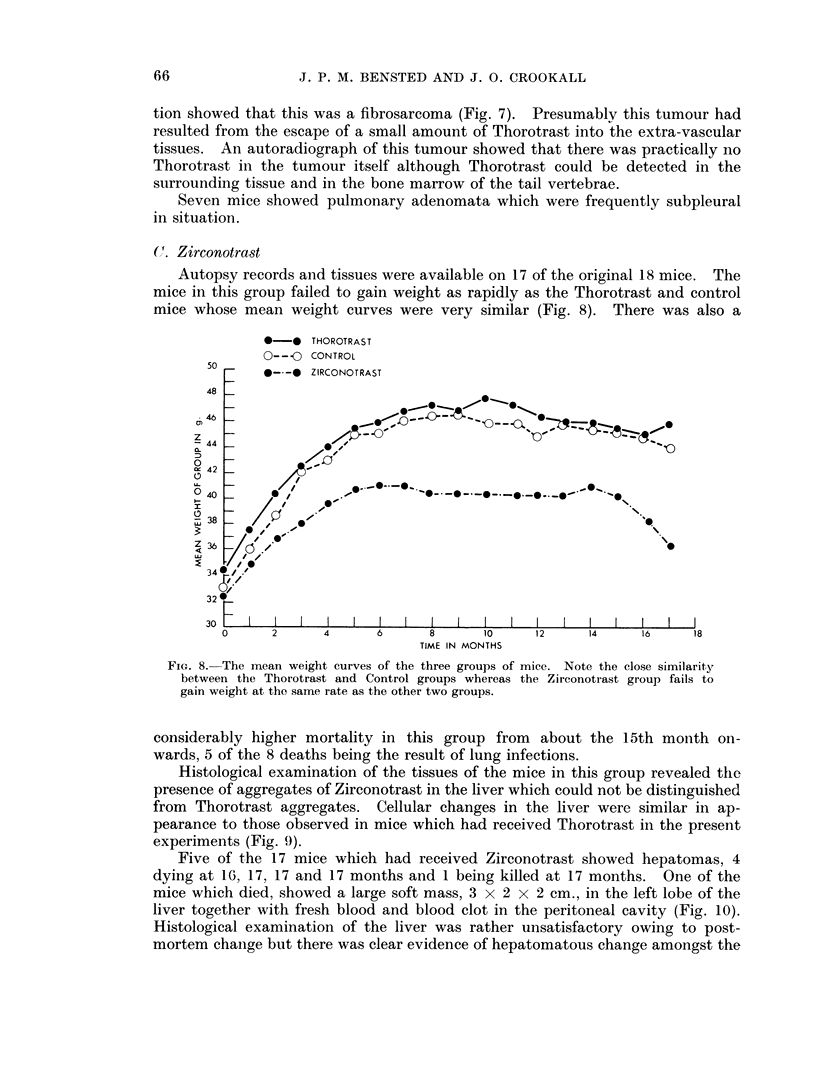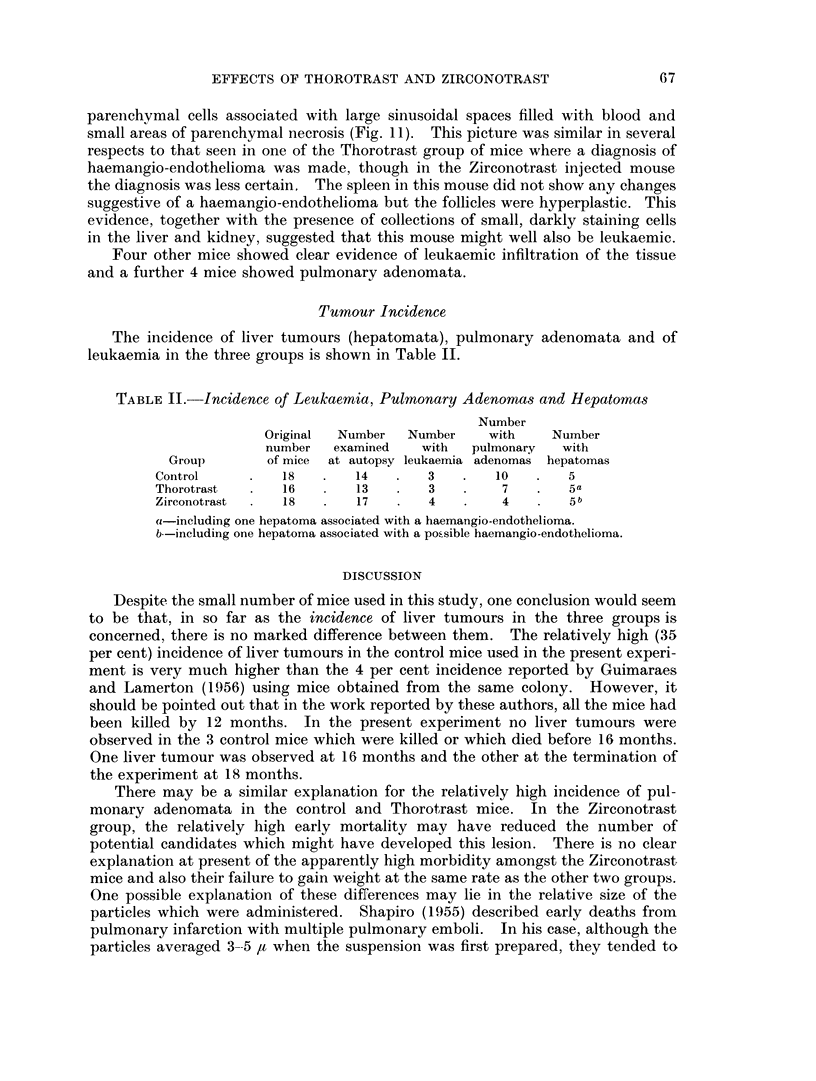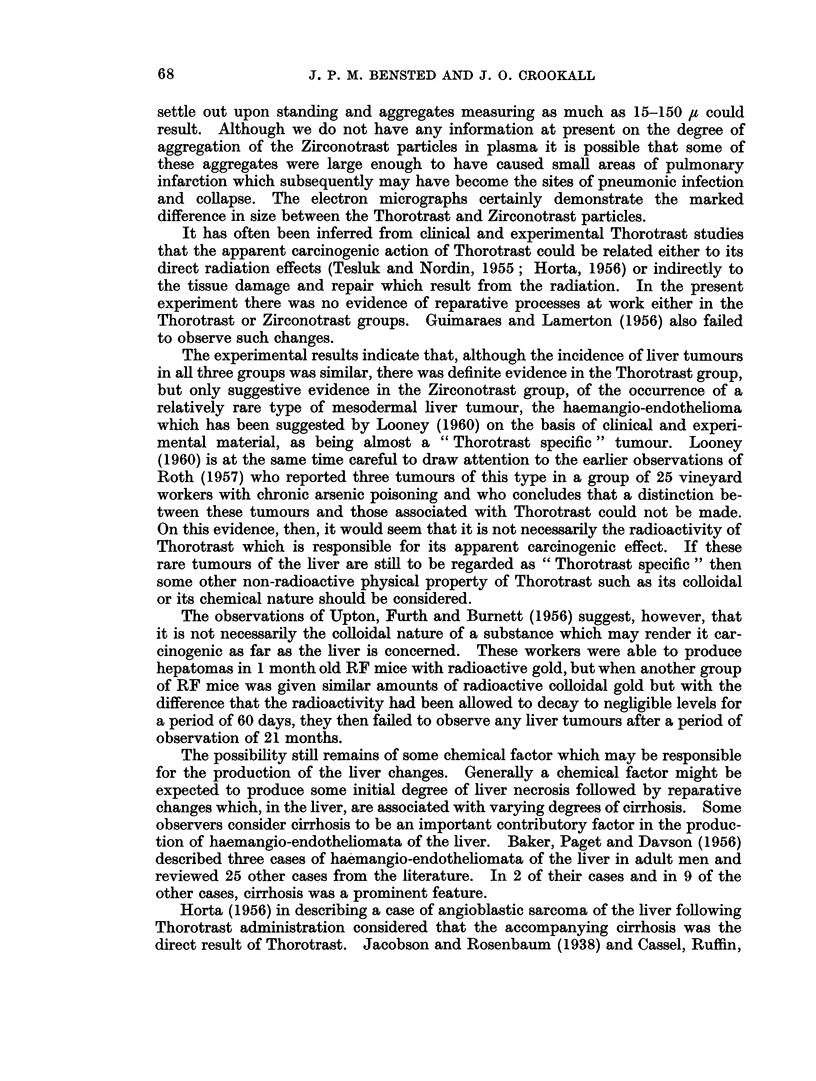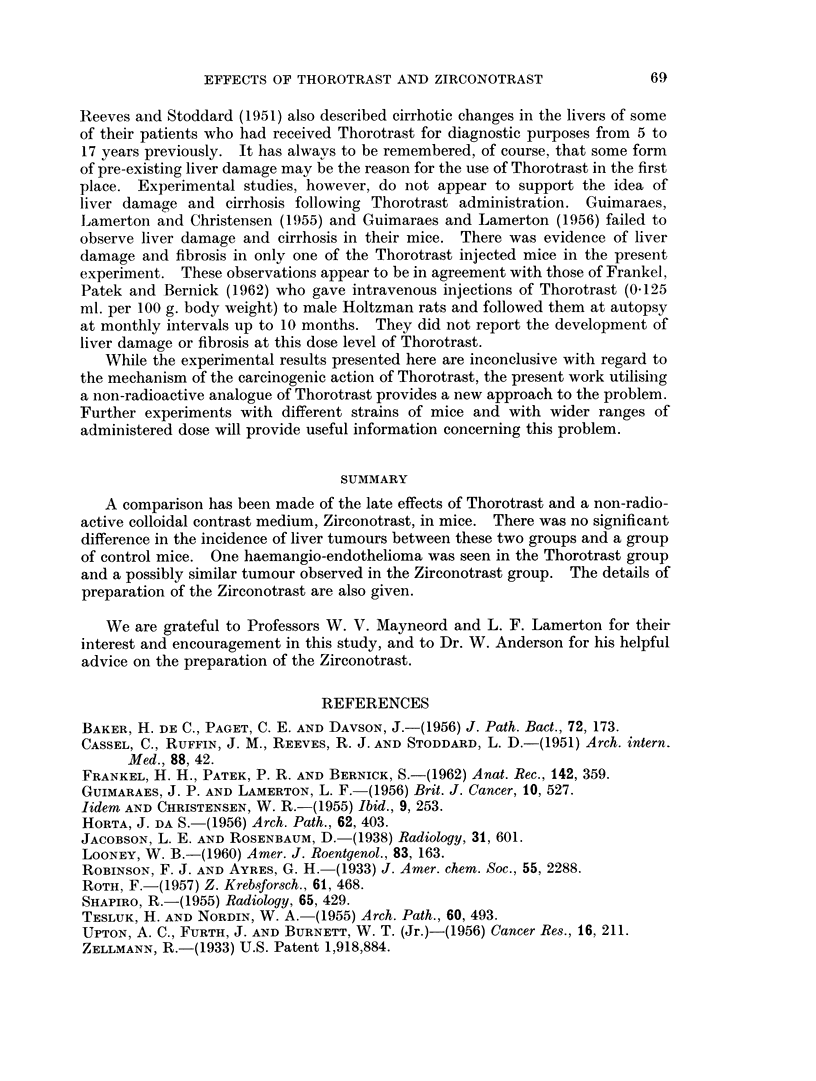# A Comparison Between the Late Effects of Thorotrast and a Non-Radioactive Zirconium Hydroxide Sol in Mice

**DOI:** 10.1038/bjc.1963.10

**Published:** 1963-03

**Authors:** J. P. M. Bensted, J. O. Crookall

## Abstract

**Images:**


### A COMPARISON BETWEEN THE LATE EFFECTS OF THOROTRAST AND A NON-RADIOACTIVE ZIRCONIUM HYDROXIDE SOL IN MICE

J. P. M. BENSTED AND J. O. CROOKALL

*From The Physics Department, Institute of Cancer Research, London S.W.3*

Received for publication November 16, 1962

IN a previous communication (Guimaraes and Lamerton, 1956) upon the late effects of Thorotrast administration in mice, it was suggested that the apparent carcinogenic effect of Thorotrast might be related in some way to its physical properties rather than to its purely radioactive properties. The object of the present investigation was to follow up this suggestion by employing a colloidal contrast medium which was similar to Thorotrast in its distribution and other properties but which was at the same time entirely non-radioactive, and to compare the resulting tissue damage and tumour incidence.

The ideal product for this purpose would be derived from a non-radioactive thorium isotope, but since no such isotope is known, attention was turned to the other elements in Group IV A of the Periodic Table, and in particular to hafnium, the closest analogue of thorium. Our attempts to produce a concentrated hafnium oxide sol were only partially successful. Owing to the relative scarcity and cost of hafnium salts further work was carried out using zirconium salts since these are virtually identical from the point of view of their chemical properties.

A concentrated zirconium oxide sol was prepared (hereafter called Zirconotrast) and this material was well tolerated when given to mice by intravenous injection. This contrasts with a report (Shapiro, 1955) that whereas soluble zirconium salts are relatively non-toxic, a colloidal zirconium oxide preparation consistently caused death from pulmonary infarction when given intravenously to rabbits and guinea-pigs. In view of this finding we thought it worth while to include details of our method of preparing Zirconotrast.

The present article concerns the results of a comparative study of the effects resulting from intravenous injection of Thorotrast and Zirconotrast into small groups of mice which were observed radiologically over a period of time and finally killed 8–17 months later for histological studies.

### METHODS

### *Zirconotrast*

The preparative scheme consists essentially of three parts. First, formation of a dilute zirconium oxide sol by hydrolysis at the boiling point of a solution of zirconyl chloride (Robinson and Ayres, 1933). Second, neutralisation of the acid sol without the formation of locally high pHs which cause gelling, and third, concentration of the sol after the addition of dextrin as a stabilising agent. Dextrin was chosen since this is used in the preparation of Thorotrast (Zellmann, 1933).

Twenty g. of z'rconium oxychloride ($ZrOCl_2 . 8 H_2O$) are dissolved in about

100 ml. distilled water, filtered through paper (Whatman 531) and the volume made up to 800 ml. This solution is boiled under reflux and after approximately 25 hours the absorption at 680 m$\mu$. is measured at intervals (1 or 2 hours) using a Uvispek spectrophotometer (Hilger and Watts). When the absorption reaches a density of 0·45 for a 1 cm. light path (*circa* 30 hours) boiling is stopped. On standing, a small amount of fine particulate matter settles out, but the sol is stable for at least two weeks, as indicated by constant absorption readings during this time. 400 ml. of this stock sol, pH 1·2, is then evaporated down to 200 ml., allowed to cool and 200 ml. of a weak base anion exchange resin (De-Acidite G—Permutit Co.,) in the base form added with stirring. After 10 minutes stirring is stopped and the pH measured, with repetition at suitable intervals until a pH value of 4·7–4·8 is reached. Immediately the sol is decanted through a glass wool filter and the resin washed twice with water by decantation (100 ml. each wash), the washings being added to the main filtrate.

The sol is now stabilised by addition of a saturated dextrin solution sufficient to give a final concentration, after further evaporation, of approximately 14 per cent w/v. The dextrin solution is prepared by stirring a large excess of dextrin puriss with distilled water for 2 hours, followed by centrifugation. The supernatant is largely clear and has a dextrin concentration of approximately 13 per cent w/v. The sol with added dextrin is evaporated down to about 40 ml. with rapid stirring (stainless steel paddle), the pH now being 3·2. It should be noted that in the case of concentrated sols a constant pH reading was only obtained after at least 10 minutes immersion of the electrodes. The resin treatment outlined above is repeated, but using 15 ml. of De-Acidite G in the base form and 40 ml. of partially concentrated sol, with a mixing time of 8 minutes. The pH of the sol after filtration is 6·15 and with the washings is 6·6. 25 ml. 0·2 per cent methyl *p*-hydroxy benzoate is added as sterilising agent, and the mixture evaporated down to a final volume of 25 ml., the pH now being 6·3. The sol is finally passed through a sintered glass filter (porosity 4) and loaded into sterilised bottles.

The concentration of zirconium in the sol was determined in the following way : 0·3 ml. aliquot of the sol was boiled with 5 ml. dilute hydrochloric acid, and after dilution to 20 ml. a small excess of sodium hydroxide was added and the mixture boiled for an hour, the evaporation loss being replenished at intervals. The fine white granular precipitate was collected on filter paper (Whatman 542), washed well with water, dried and ignited in a tarred platinum dish. The weight obtained was taken as the weight of zirconium oxide in the sample aliquot.

The Zirconotrast used in the experimental work reported in this paper had the following composition :

Zirconium dioxide 13 per cent w/v
Dextrin 17 per cent w/v
Methyl *p*-hydroxy benzoate 0·2 per cent
pH 6·3

*Particle size*

Electron micrographs indicate (Fig. 1 and 2), on the assumption that the particles are spherical, that 90 per cent of the weight of zirconium oxide is contained in particles with diameters ranging from 50–100 m$\mu$. It should be noted that by comparison, Thorotrast particles are, on average, 5–6 times smaller and in addition have a more irregular and angular shape. This difference follows from the modes of preparation of the two sols. For Zirconotrast, this involved accretion of ultramicro particles in solution, and for Thorotrast, dispersion of a solid phase, thorium dioxide, obtained by thermal decomposition of thorium oxalate.

### EXPERIMENTAL

Fifty-two male Schofield mice between 30–35 g. were divided into 3 groups as follows :

Group A.—16 mice given 0·1 ml. of Thorotrast intravenously.
Group B.—18 mice given 0·1 ml. of Zirconotrast intravenously.
Group C.—18 normal, uninjected mice as controls.

All the 3 groups were maintained on Aberdeen rat cake as in the previous experiments and water was given *ad libitum*. Groups A and B were radiographed at intervals to confirm the presence and persistence of the contrast materials.

There were no immediate deaths following the injection of the Thorotrast or the Zirconotrast. The first death occurred from broncho-pneumonia in the Zirconotrast group at 8 months. The majority of the mice in all 3 groups survived for over a year. The experiment was terminated at 18 months by killing all the remaining animals. These mice were autopsied and histological studies made of the liver, spleen, lungs, kidneys, lymph nodes, testis and in some cases, the bone marrow. The number of survivors in each group at the end of the experiment is indicated in Table I.

---

### EXPLANATION OF PLATES

FIG. 1.—Electron micrograph of Zirconotrast particles, magnification × 50,000. By courtesy of Dr. E. H. Mercer, Chester Beatty Research Institute, London, S.W.3.

FIG. 2.—Electron micrograph of Thorotrast particles, magnification × 50,000. By courtesy of Dr. E. H. Mercer, Chester Beatty Research Institute, London, S.W.3.

FIG. 3.—A small superficial hepatoma in the liver of a 16 month old, control mouse. H & E × 20.

FIG. 4.—Histological appearance of the liver of a mouse given 0·1 ml. Thorotrast 17 months previously, showing a hepatoma (right half of field). Note the Thorotrast aggregates in the normal liver whereas the hepatoma contains none. Also the absence of any form of tissue reaction. H. & E. × 20.

FIG. 5.—The appearance of the liver of a mouse 17 months after a dose of 0·1 ml. Thorotrast. Part of a large, soft and haemorrhagic tumour, 2·3 × 2·0 × 1·2 cm. occupying the left lobe. The Thorotrast aggregates are confined to one area of the liver. The remainder consists of abnormally orientated liver cords with areas of necrosis and numerous blood-filled spaces, several of which contain ante-mortem thrombus. H. & E. × 12.

FIG. 6.—Radiograph of the mouse in Fig. 5. Note the absence of Thorotrast from the centre of the liver tumour.

FIG. 7.—Subcutaneous sarcoma on the tail of a mouse given an intravenous injection of 0·1 ml. Thorotrast in this region 13 months previously. Autoradiography of this tumour showed alpha-tracks only at the edge of the tumour in the region of macrophages. Some tracks were present in the bone marrow. H. & E. × 15.

FIG. 9.—The margin between an area of normal liver, to the right, and a small hepatoma in a mouse 17 months after injection of 0·1 ml. of the Zirconotrast preparation. Note the Zirconotrast aggregates and the absence of reaction around them. H. & E. × 105.

FIG. 10.—Radiograph of the mouse whose liver tumour is depicted in Fig. 11.

FIG. 11.—Part of a large, soft haemorrhagic tumour, 3 × 2 cm., in the left lobe of the liver of a mouse which died 17 months after receiving 0·1 ml. Zirconotrast. At autopsy a large amount of blood and blood clot was present in the peritoneal cavity. Note the large, blood-filled spaces similar to those seen in Fig. 5. The remainder of the section shows areas of hepatomatous change. H. & E. × 18.

1

2

3

4

5

6

Bensted and Crookall.

7

9

10

11

TABLE I.—*Survival of Experimental Mice*

| Group | Original number of mice | Number of survivors at termination of experiment | Per cent survivors |
|---|---|---|---|
| Thorotrast | 16 | 6 | 37 |
| Zirconotrast | 18 | 4 | 22 |
| Control | 18 | 9 | 50 |

*Histological Observations*

*A. Control*

Autopsy records and tissues were available on 14 of the original 18 mice. Five of the 18 mice showed liver tumours (Fig. 3) and 10 showed pulmonary adenomata. Three of the 18 mice showed leukaemic infiltration of the tissues.

*B. Thorotrast group*

Autopsy records and tissues were available on 13 out of the original 16 mice. In the liver, the histological changes were similar to those described previously (Guimaraes, Lamerton and Christensen, 1955: Guimaraes and Lamerton, 1956). The Thorotrast aggregates frequently abutted onto a hepatic vein into which they could very readily have ruptured, thus affording a means whereby the Thorotrast could be further distributed throughout the body at a late interval after the original injection.

In this group there were 5 liver tumours which were classified as hepatomata, one found at 12 months and four at 17 months at the termination of the experiment (Fig. 4). One of these latter mice, whose liver contained a large, soft, haemorrhagic tumour, $2{\cdot}3 \times 2 \times 1{\cdot}2$ cm., showed histological changes suggestive of a haemangio-endothelioma of the liver. (Fig. 5). These changes consisted of numerous large irregular spaces, some filled with blood cells and others with a reticular meshwork of eosinophilic, amorphous material. Those spaces which contained blood could be seen to possess a definite endothelial lining but without any marked proliferative changes being evident. In some of them ante-mortem thrombus material was present. There were also some areas of parenchymal necrosis and interstitial fibrosis. In this case, the spleen was slightly enlarged and on histological examination, showed several large blood filled spaces some of which contained ante-mortem thrombus. The interpretation of the structure of the endothelial lining of these spaces in the spleen was difficult owing to post-mortem change. The histological changes in the liver and spleen were very similar to those which have been described in cases of haemangio-endothelioma (Roth, 1957). In this particular case the most malignant changes appeared to be in the spleen in which organ the tumour may be regarded as originating. Fig. 6 is a post-mortem radiograph of the mouse showing the Thorotrast present in the spleen and liver but only in the periphery of the latter.

Three examples of a lymphatic type of leukaemia were observed, characterised by enlargement of the spleen and lymph nodes and with extensive leukaemic infiltration of other organs. One of these leukaemias was present in a mouse with a hepatoma.

One mouse, killed at 13 months, showed a firm subcutaneous swelling of the tail at the site of the intravenous injection of Thorotrast. Histological examination showed that this was a fibrosarcoma (Fig. 7). Presumably this tumour had resulted from the escape of a small amount of Thorotrast into the extra-vascular tissues. An autoradiograph of this tumour showed that there was practically no Thorotrast in the tumour itself although Thorotrast could be detected in the surrounding tissue and in the bone marrow of the tail vertebrae.

Seven mice showed pulmonary adenomata which were frequently subpleural in situation.

*C. Zirconotrast*

Autopsy records and tissues were available on 17 of the original 18 mice. The mice in this group failed to gain weight as rapidly as the Thorotrast and control mice whose mean weight curves were very similar (Fig. 8). There was also a

FIG. 8.—The mean weight curves of the three groups of mice. Note the close similarity between the Thorotrast and Control groups whereas the Zirconotrast group fails to gain weight at the same rate as the other two groups.

considerably higher mortality in this group from about the 15th month onwards, 5 of the 8 deaths being the result of lung infections.

Histological examination of the tissues of the mice in this group revealed the presence of aggregates of Zirconotrast in the liver which could not be distinguished from Thorotrast aggregates. Cellular changes in the liver were similar in appearance to those observed in mice which had received Thorotrast in the present experiments (Fig. 9).

Five of the 17 mice which had received Zirconotrast showed hepatomas, 4 dying at 16, 17, 17 and 17 months and 1 being killed at 17 months. One of the mice which died, showed a large soft mass, 3 × 2 × 2 cm., in the left lobe of the liver together with fresh blood and blood clot in the peritoneal cavity (Fig. 10). Histological examination of the liver was rather unsatisfactory owing to post-mortem change but there was clear evidence of hepatomatous change amongst the parenchymal cells associated with large sinusoidal spaces filled with blood and small areas of parenchymal necrosis (Fig. 11). This picture was similar in several respects to that seen in one of the Thorotrast group of mice where a diagnosis of haemangio-endothelioma was made, though in the Zirconotrast injected mouse the diagnosis was less certain. The spleen in this mouse did not show any changes suggestive of a haemangio-endothelioma but the follicles were hyperplastic. This evidence, together with the presence of collections of small, darkly staining cells in the liver and kidney, suggested that this mouse might well also be leukaemic.

Four other mice showed clear evidence of leukaemic infiltration of the tissue and a further 4 mice showed pulmonary adenomata.

### *Tumour Incidence*

The incidence of liver tumours (hepatomata), pulmonary adenomata and of leukaemia in the three groups is shown in Table II.

TABLE II.—*Incidence of Leukaemia, Pulmonary Adenomas and Hepatomas*

| Group | Original number of mice | Number examined at autopsy | Number with leukaemia | Number with pulmonary adenomas | Number with hepatomas |
|---|---|---|---|---|---|
| Control | 18 | 14 | 3 | 10 | 5 |
| Thorotrast | 16 | 13 | 3 | 7 | 5[a] |
| Zirconotrast | 18 | 17 | 4 | 4 | 5[b] |

a—including one hepatoma associated with a haemangio-endothelioma.
b—including one hepatoma associated with a possible haemangio-endothelioma.

### DISCUSSION

Despite the small number of mice used in this study, one conclusion would seem to be that, in so far as the *incidence* of liver tumours in the three groups is concerned, there is no marked difference between them. The relatively high (35 per cent) incidence of liver tumours in the control mice used in the present experiment is very much higher than the 4 per cent incidence reported by Guimaraes and Lamerton (1956) using mice obtained from the same colony. However, it should be pointed out that in the work reported by these authors, all the mice had been killed by 12 months. In the present experiment no liver tumours were observed in the 3 control mice which were killed or which died before 16 months. One liver tumour was observed at 16 months and the other at the termination of the experiment at 18 months.

There may be a similar explanation for the relatively high incidence of pulmonary adenomata in the control and Thorotrast mice. In the Zirconotrast group, the relatively high early mortality may have reduced the number of potential candidates which might have developed this lesion. There is no clear explanation at present of the apparently high morbidity amongst the Zirconotrast mice and also their failure to gain weight at the same rate as the other two groups. One possible explanation of these differences may lie in the relative size of the particles which were administered. Shapiro (1955) described early deaths from pulmonary infarction with multiple pulmonary emboli. In his case, although the particles averaged 3–5 $\mu$ when the suspension was first prepared, they tended to settle out upon standing and aggregates measuring as much as 15–150 $\mu$ could result. Although we do not have any information at present on the degree of aggregation of the Zirconotrast particles in plasma it is possible that some of these aggregates were large enough to have caused small areas of pulmonary infarction which subsequently may have become the sites of pneumonic infection and collapse. The electron micrographs certainly demonstrate the marked difference in size between the Thorotrast and Zirconotrast particles.

It has often been inferred from clinical and experimental Thorotrast studies that the apparent carcinogenic action of Thorotrast could be related either to its direct radiation effects (Tesluk and Nordin, 1955; Horta, 1956) or indirectly to the tissue damage and repair which result from the radiation. In the present experiment there was no evidence of reparative processes at work either in the Thorotrast or Zirconotrast groups. Guimaraes and Lamerton (1956) also failed to observe such changes.

The experimental results indicate that, although the incidence of liver tumours in all three groups was similar, there was definite evidence in the Thorotrast group, but only suggestive evidence in the Zirconotrast group, of the occurrence of a relatively rare type of mesodermal liver tumour, the haemangio-endothelioma which has been suggested by Looney (1960) on the basis of clinical and experimental material, as being almost a "Thorotrast specific" tumour. Looney (1960) is at the same time careful to draw attention to the earlier observations of Roth (1957) who reported three tumours of this type in a group of 25 vineyard workers with chronic arsenic poisoning and who concludes that a distinction between these tumours and those associated with Thorotrast could not be made. On this evidence, then, it would seem that it is not necessarily the radioactivity of Thorotrast which is responsible for its apparent carcinogenic effect. If these rare tumours of the liver are still to be regarded as "Thorotrast specific" then some other non-radioactive physical property of Thorotrast such as its colloidal or its chemical nature should be considered.

The observations of Upton, Furth and Burnett (1956) suggest, however, that it is not necessarily the colloidal nature of a substance which may render it carcinogenic as far as the liver is concerned. These workers were able to produce hepatomas in 1 month old RF mice with radioactive gold, but when another group of RF mice was given similar amounts of radioactive colloidal gold but with the difference that the radioactivity had been allowed to decay to negligible levels for a period of 60 days, they then failed to observe any liver tumours after a period of observation of 21 months.

The possibility still remains of some chemical factor which may be responsible for the production of the liver changes. Generally a chemical factor might be expected to produce some initial degree of liver necrosis followed by reparative changes which, in the liver, are associated with varying degrees of cirrhosis. Some observers consider cirrhosis to be an important contributory factor in the production of haemangio-endotheliomata of the liver. Baker, Paget and Davson (1956) described three cases of haemangio-endotheliomata of the liver in adult men and reviewed 25 other cases from the literature. In 2 of their cases and in 9 of the other cases, cirrhosis was a prominent feature.

Horta (1956) in describing a case of angioblastic sarcoma of the liver following Thorotrast administration considered that the accompanying cirrhosis was the direct result of Thorotrast. Jacobson and Rosenbaum (1938) and Cassel, Ruffin,

Reeves and Stoddard (1951) also described cirrhotic changes in the livers of some of their patients who had received Thorotrast for diagnostic purposes from 5 to 17 years previously. It has always to be remembered, of course, that some form of pre-existing liver damage may be the reason for the use of Thorotrast in the first place. Experimental studies, however, do not appear to support the idea of liver damage and cirrhosis following Thorotrast administration. Guimaraes, Lamerton and Christensen (1955) and Guimaraes and Lamerton (1956) failed to observe liver damage and cirrhosis in their mice. There was evidence of liver damage and fibrosis in only one of the Thorotrast injected mice in the present experiment. These observations appear to be in agreement with those of Frankel, Patek and Bernick (1962) who gave intravenous injections of Thorotrast (0·125 ml. per 100 g. body weight) to male Holtzman rats and followed them at autopsy at monthly intervals up to 10 months. They did not report the development of liver damage or fibrosis at this dose level of Thorotrast.

While the experimental results presented here are inconclusive with regard to the mechanism of the carcinogenic action of Thorotrast, the present work utilising a non-radioactive analogue of Thorotrast provides a new approach to the problem. Further experiments with different strains of mice and with wider ranges of administered dose will provide useful information concerning this problem.

### SUMMARY

A comparison has been made of the late effects of Thorotrast and a non-radioactive colloidal contrast medium, Zirconotrast, in mice. There was no significant difference in the incidence of liver tumours between these two groups and a group of control mice. One haemangio-endothelioma was seen in the Thorotrast group and a possibly similar tumour observed in the Zirconotrast group. The details of preparation of the Zirconotrast are also given.

We are grateful to Professors W. V. Mayneord and L. F. Lamerton for their interest and encouragement in this study, and to Dr. W. Anderson for his helpful advice on the preparation of the Zirconotrast.

### REFERENCES

Baker, H. de C., Paget, C. E. and Davson, J.—(1956) *J. Path. Bact.*, **72**, 173.
Cassel, C., Ruffin, J. M., Reeves, R. J. and Stoddard, L. D.—(1951) *Arch. intern. Med.*, **88**, 42.
Frankel, H. H., Patek, P. R. and Bernick, S.—(1962) *Anat. Rec.*, **142**, 359.
Guimaraes, J. P. and Lamerton, L. F.—(1956) *Brit. J. Cancer*, **10**, 527.
*Iidem* and Christensen, W. R.—(1955) *Ibid.*, **9**, 253.
Horta, J. da S.—(1956) *Arch. Path.*, **62**, 403.
Jacobson, L. E. and Rosenbaum, D.—(1938) *Radiology*, **31**, 601.
Looney, W. B.—(1960) *Amer. J. Roentgenol.*, **83**, 163.
Robinson, F. J. and Ayres, G. H.—(1933) *J. Amer. chem. Soc.*, **55**, 2288.
Roth, F.—(1957) *Z. Krebsforsch.*, **61**, 468.
Shapiro, R.—(1955) *Radiology*, **65**, 429.
Tesluk, H. and Nordin, W. A.—(1955) *Arch. Path.*, **60**, 493.
Upton, A. C., Furth, J. and Burnett, W. T. (Jr.)—(1956) *Cancer Res.*, **16**, 211.
Zellmann, R.—(1933) U.S. Patent 1,918,884.